# Supplementary material for: Circular RNA circGlis3 protects against islet β-cell dysfunction and apoptosis in obesity
Source: Nat Commun. 2023 Jan 21;14:351. doi: 10.1038/s41467-023-35998-z (PMC9867769; doi:10.1038/s41467-023-35998-z)
Supplement: Supplementary file 1 — Supplementary Information [file 41467_2023_35998_MOESM1_ESM.pdf]

Supplementary Information:

Circular RNA circGlis3 protects against islet β-cell dysfunction and apoptosis in obesity

Yue Liu[1], Yue Yang[1], Chenying Xu[1], Jianxing Liu[1], Jiale Chen[1], Guoqing Li[2], Bin Huang[1], Yi Pan[1], Yanfeng Zhang[1], Qiong Wei[2], Stephen J Pandol[3], Fangfang Zhang[1,*], Ling Li[2,*], Liang Jin[1,*]

File list

Supplementary Figures:

Supplementary Fig. 1

Supplementary Fig. 2

Supplementary Fig. 3

Supplementary Fig. 4

Supplementary Fig. 5

Supplementary Fig. 6

Supplementary Fig. 7

Supplementary Table 1

Supplementary Table 2

Supplementary Figures:

Supplementary Fig. 1

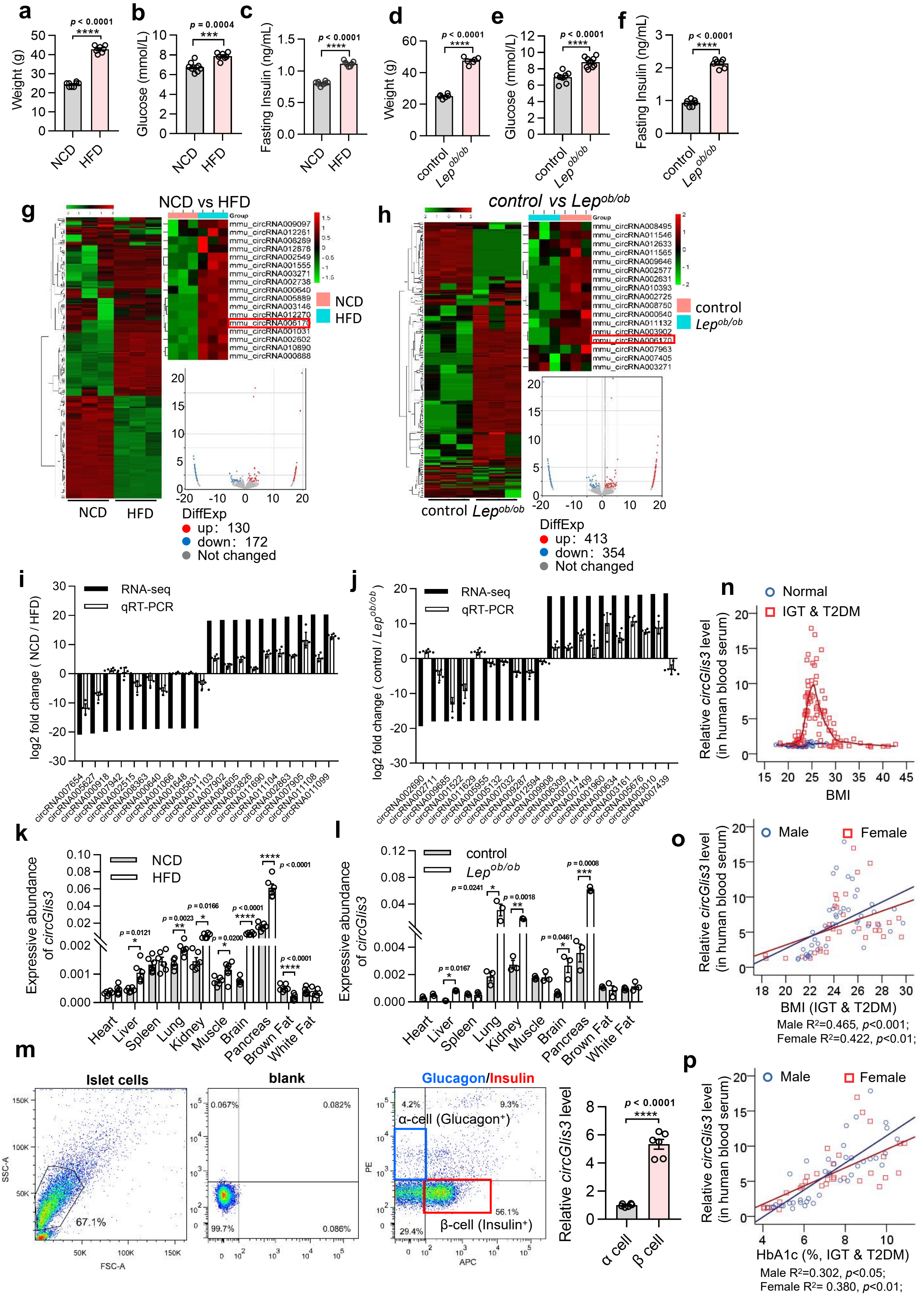

Supplementary Fig. 1 circGlis3 is upregulated in and is associated with obesity, Related to Fig. 1.

a-c. The body weight, blood glucose and insulin levels of HFD-fed mice (n = 9 biological animals). **d-f.** The body weight, blood glucose and insulin levels of $Lep^{ob/ob}$ mice (n = 6 biological animals). **g.** Heatmap and volcano pot showing circRNAs upregulated and downregulated differentially in islets of HFD-fed mice and NCD-fed mice (n = 3 biological subjects, log2-fold change >2 or <-2, $p < 0.05$). **h.** Heatmap and volcano pot showing circRNAs upregulated and downregulated differentially in islets of $Lep^{ob/ob}$ mice and control mice (n = 3 biological subjects, log2-fold change >2 or <-2, $p < 0.05$). **i.** RT-PCR analysis of the 20 top dysregulated circRNAs in islets of the NCD- and HFD-fed mice (n = 6 biological animals). **j.** RT-PCR analysis of the 20 top dysregulated circRNAs in islets of the $Lep^{ob/ob}$ mice and control mice (n = 6 biological animals). **k-l.** The expressive abundance of circGlis3 in various tissues of the HFD-fed mice and $Lep^{ob/ob}$ mice (n = 6 biological animals). **m.** β-cell and α-cell sorting and measurement of the level of circGlis3 by using RT-PCR (n = 6 biological replicates). **n.** circGlis3 expression in the serum of human individuals with obesity and moderate diabetes (Normal n = 29, IGT and/or T2DM n = 89). **o-p.** The correlation analysis was applied between the level of circGlis3 and BMI (BMI < 30, IGT and/or T2DM n = 75; Male $R^2 = 0.465$, $p < 0.001$; Female $R^2 = 0.422$, $p < 0.01$), and HbA1c (IGT and/or T2DM n = 75; Male $R^2 = 0.302$, $p < 0.05$; Female $R^2 = 0.380$, $p < 0.05$). **a-f, i-m** For bar and line graphs, data represents mean ± SEM. **a-f, k-m** Unpaired two-tailed Student's t-test. **n** LOESS Curve Fitting, 35% of points to fit. **o-p** Pearson correlation and regression analysis. $^{*}p < 0.05$, $^{**}p < 0.01$, $^{***}p < 0.001$, $^{****}p < 0.0001$. Source data are provided as a Source data file.

Supplementary Fig. 2

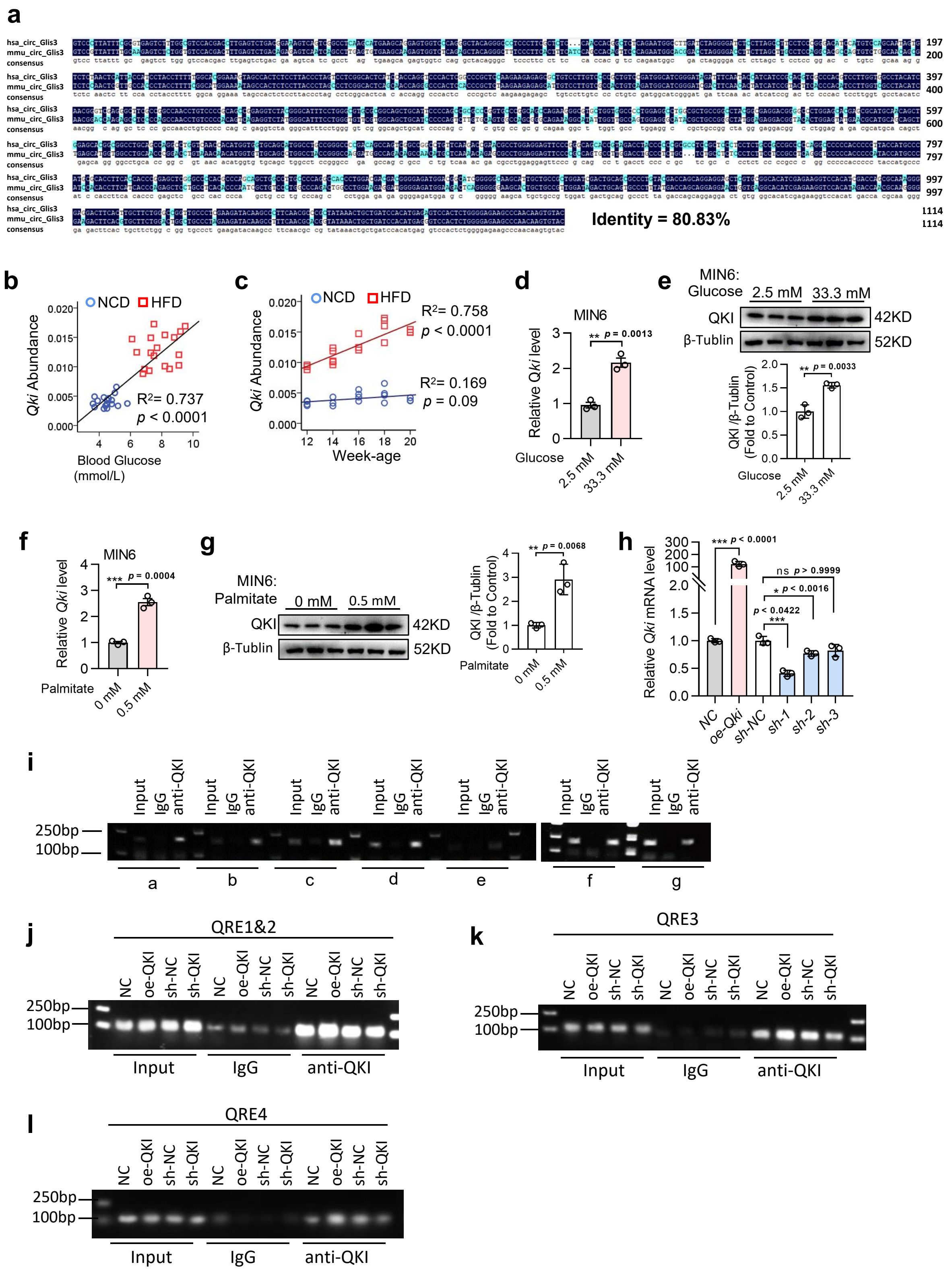

Supplementary Fig. 2 Identification of circGlis3, and splicing factor QKI regulates formation of circGlis3, Related to Fig. 2.

a. Homologous analysis of the human circGlis3 and mouse circGlis3. **b.** The correlation of fasting blood glucose and *Qki* mRNA abundance in the islets of the NCD- and HFD-fed mice (n = 18 biological animals). **c.** The correlation of week-age and *Qki* mRNA abundance in islets from NCD- and HFD-fed mice (n = 18 biological animals). **d-e.** RT-PCR analysis and Western Blotting showing *Qki* mRNA and protein expression in glucose (2.5 mM and 33.3 mM) stimulated MIN6 cells (n = 3 biological replicates). **f-g.** RT-PCR analysis and Western Blotting showing *Qki* mRNA and protein expression in palmitate (0 mM and 0.5 mM) stimulated MIN6 cells (n = 3 biological replicates). **h.** Expression efficiency of *Qki* mRNA in MIN6 cells (n = 3 biological replicates). **i.** DNA gel electrophoresis of RIP and RT-PCR analysis shown in Fig. 2m. **j-l.** DNA gel electrophoresis of RIP and RT-PCR analysis shown in Fig. 2o-q. **d-h** For bar and line graphs, data represents mean ± SEM. **b-c** Pearson correlation and regression analysis. **d-g** Unpaired two-tailed Student's t-test. **h** One-way ANOVA with Tukey's post-test. $^{*}p < 0.05$, $^{**}p < 0.01$, $^{***}p < 0.001$, $^{****}p < 0.0001$. Source data are provided as a Source data file.

Supplementary Fig. 3

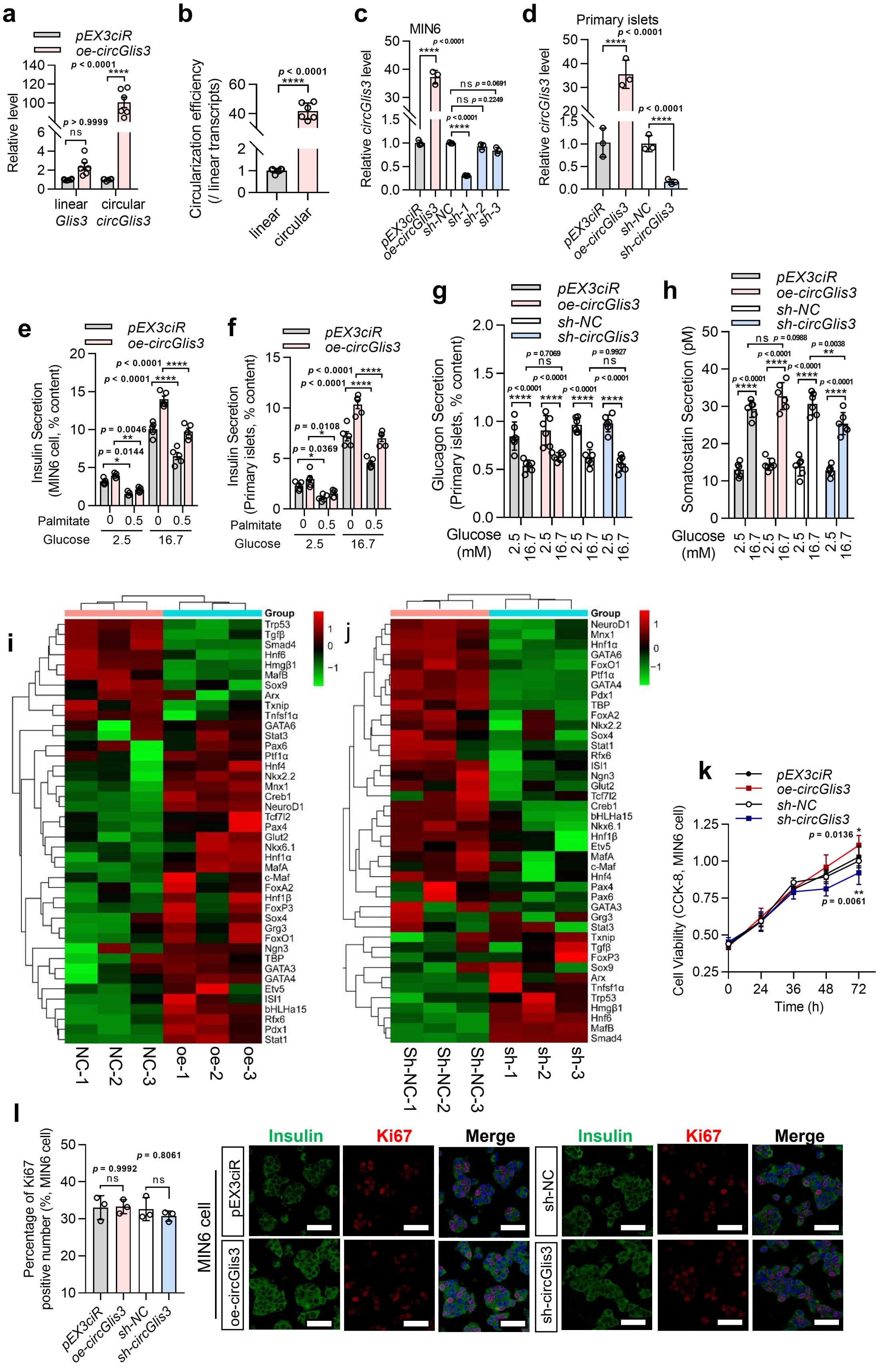

Supplementary Fig. 3 Upregulation of circGlis3 promotes insulin transcription and secretion, and inhibits β-cell apoptosis *in vitro*, Related to Fig. 3.

a-b. To assess the circularization efficiency, the level of the linear and the circular transcripts in MIN6 cells were detected by RT-PCR (n = 6 biological replicates). **c.** Expression efficiency of circGlis3 in MIN6 cells transfected with circGlis3-vector and three independent shRNAs (n = 3 biological replicates). **d.** Expression efficiency of circGlis3 in mouse islets transfected with circGlis3-vector and shRNA (n = 3 biological replicates). **e-f.** MIN6 cells (n = 5 biological replicates) and mouse islets (n = 5 biological subjects) were incubated with palmitate (0.5 mM) for 48 h prior to incubation with glucose (2.5 mM or 16.7 mM) for 2 h. Insulin secretion were then assessed as described in the methods. **g.** Glucagon secretion was measured in glucose-stimulated islet (n = 6 biological subjects). **h.** Somatostatin secretion was measured in glucose-stimulated islet (n = 6 biological subjects). **i-j.** Heatmap showing mRNA expression of 42 transcription factors in the islets of oe-circGlis3-treated and *sh*-circGlis3-treated mice (n = 3 biological subjects). **k.** CCK-8 assay of MIN6 cell with circGlis3 overexpression and knockdown (n = 6 biological replicates). **l.** Ki67 Immunofluorescent staining of MIN6 cell with circGlis3 overexpression and knockdown (Red represents positive Ki67 cells, Green represents Insulin; Scale bars represent 50 μm). **a-h, k-l** For bar and line graphs, data represents mean ± SEM. **b** Unpaired two-tailed Student's t-test. **a, e-h** Two-way ANOVA with Bonferroni's post-test. **c-d, k-l** One-way ANOVA with Tukey's post-test. $^{*}p < 0.05$, $^{**}p < 0.01$, $^{***}p < 0.001$, $^{****}p < 0.0001$. Source data are provided as a Source data file.

Supplementary Fig. 4

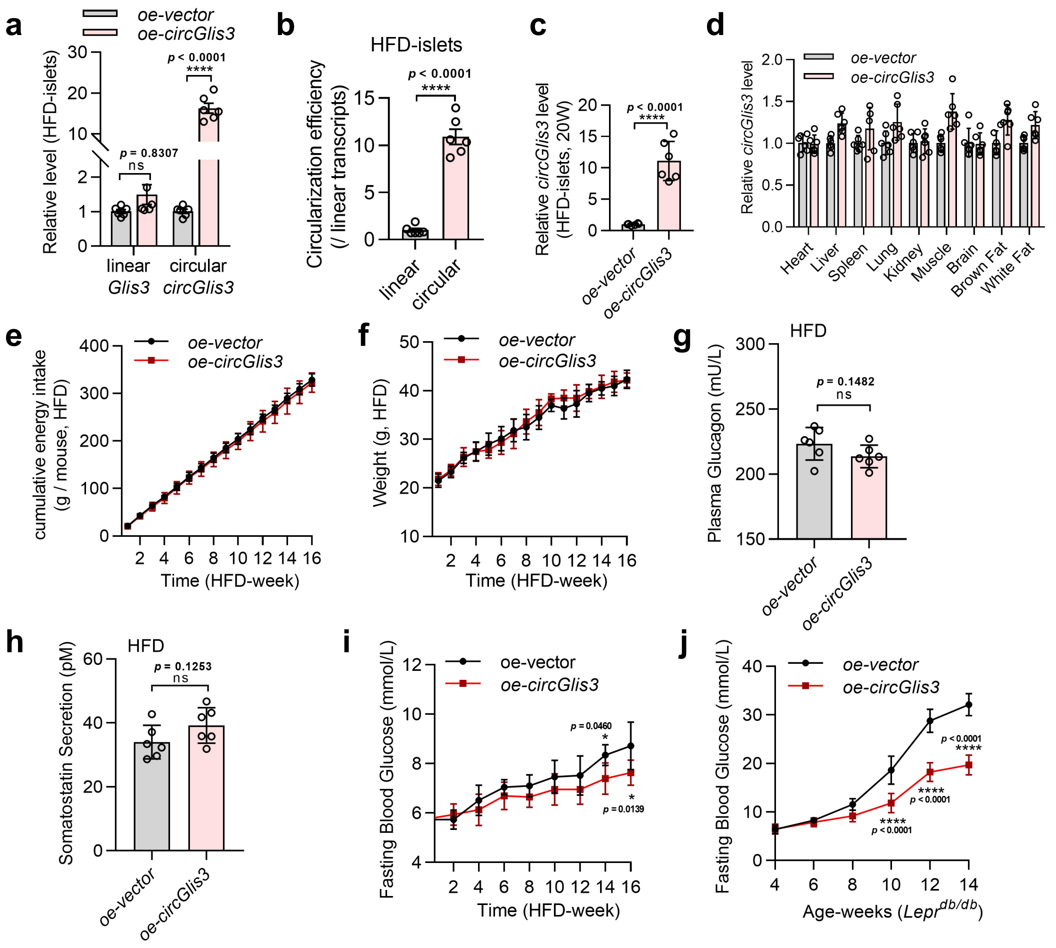

Supplementary Fig. 4 Overexpression of circGlis3 protects against β-cell dysfunction and apoptosis *in vivo*, Related to Fig. 4.

a. a-b. The circularization efficiency *in vivo* was also assessed by RT-PCR (*n* = 6 biological animals). **c.** Expression efficiency of circGlis3 in the islets of the oe-circGlis3-treated mice with HFD-fed for 20 weeks (*n* = 6 biological animals). **d.** RT-PCR analysis of circGlis3 expression in various tissues (*n* = 6 biological animals). **e-f.** Cumulative energy intake and body weight of HFD-fed mice (*n* = 6 biological animals). **g.** The glucagon levels in fasting serum of HFD-fed mice (*n* = 6 biological subjects). **h.** Glucose-stimulated (16.7 mM) somatostatin secretion in islets from HFD-fed mice (*n* = 6 biological subjects). **i.** Fasting blood glucose in negative control and oe-circGlis3-treated mice until HFD-fed for 16 weeks (*n* = 6 biological animals). **j.** Fasting blood glucose in the negative control and oe-circGlis3-treated $Lepr^{db/db}$ mice at age from 4 to 12 weeks (*n* = 6 biological animals). **a-j** For bar and line graphs, data represents mean ± SEM. **a, e-f, i-j** Two-way ANOVA with Bonferroni's post-test. **b-d, g-h** Unpaired two-tailed Student's t-test. $^{*}p < 0.05$, $^{**}p < 0.01$, $^{***}p < 0.001$, $^{****}p < 0.0001$. Source data are provided as a Source data file.

Supplementary Fig. 5

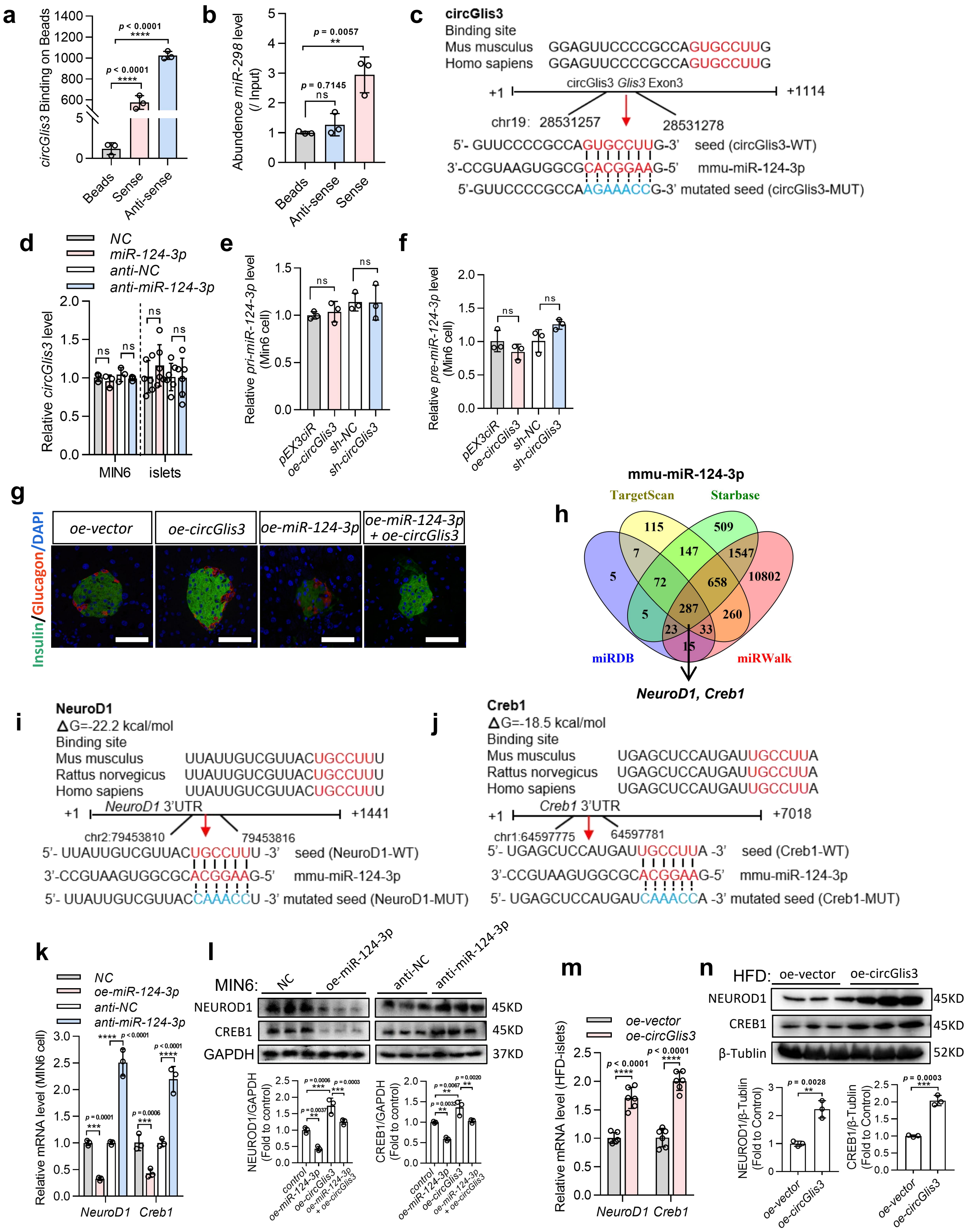

Supplementary Fig. 5 circGlis3 regulates insulin transcription by targeting and sponging miR-124-3p, Related to Fig. 5.

a. a. Biotin-labelled cyclized circGlis3 sense or anti-sense which subjected to RNA pulldown were efficiently bound on beads and measured by RT-PCR (n = 3 biological replicates). **b.** RNA pulldown and RT-PCR assay showing the amount of endogenous circGlis3 pulled down by biotin-labelled miR-298-5p (n = 6 biological replicates) (n = 3 biological replicates). **c.** Schematic representation of the constructs used in the luciferase assay. The sequences indicated the putative miR-124-3p target sites on the circGlis3-WT and circGlis3-MUT. **d.** RT-PCR assay of circGlis3 expression in MIN6 cell (n = 3 biological replicates) and islets (n = 6 biological subjects) with transfecting miR-124-3p mimics and inhibitors. **e-f.** RT-PCR assay of pri-miR-124-3p *and* pre-miR-124-3p expression in MIN6 cells (n = 3 biological replicates) and islets (n = 6 biological subjects) with circGlis3 overexpression and knockdown. **g.** Insulin and glucagon immunofluorescent staining in the islets from oe-miR-124-3p or/and *oe*-circGlis3 treated mice (Red represents Glucogan, Green represents Insulin; Scale bars represent 100 μm). **h.** Schematic illustration showing the overlap of the target mRNAs of miR-124-3p predicted by TargetScan, StarBase, miRDB, and miRWalk. **i-j.** Schematic of *NeuroD1* and *Creb1* 3’-UTR WT and MUT luciferase reporter vectors were shown. **k-l.** *NeuroD1* and *Creb1* mRNA and protein expression levels in MIN6 cells with transfecting miR-124-3p mimics and inhibitors (n = 3 biological replicates). **m-n.** *NeuroD1* and *Creb1* mRNA and protein expression levels in the islets from *oe*-circGlis3-treated mice (n = 6 biological subjects). **a-b, d-f, k-n** For bar graphs, data represents mean $\pm$ SEM. **a-b, d-f, l-n** One-way ANOVA with Tukey’s post-test. **k, m** Two-way ANOVA with Bonferroni’s post-test. $^{*}p < 0.05$, $^{**}p < 0.01$, $^{***}p < 0.001$, $^{****}p < 0.0001$. Source data are provided as a Source data file.

Supplementary Fig. 6

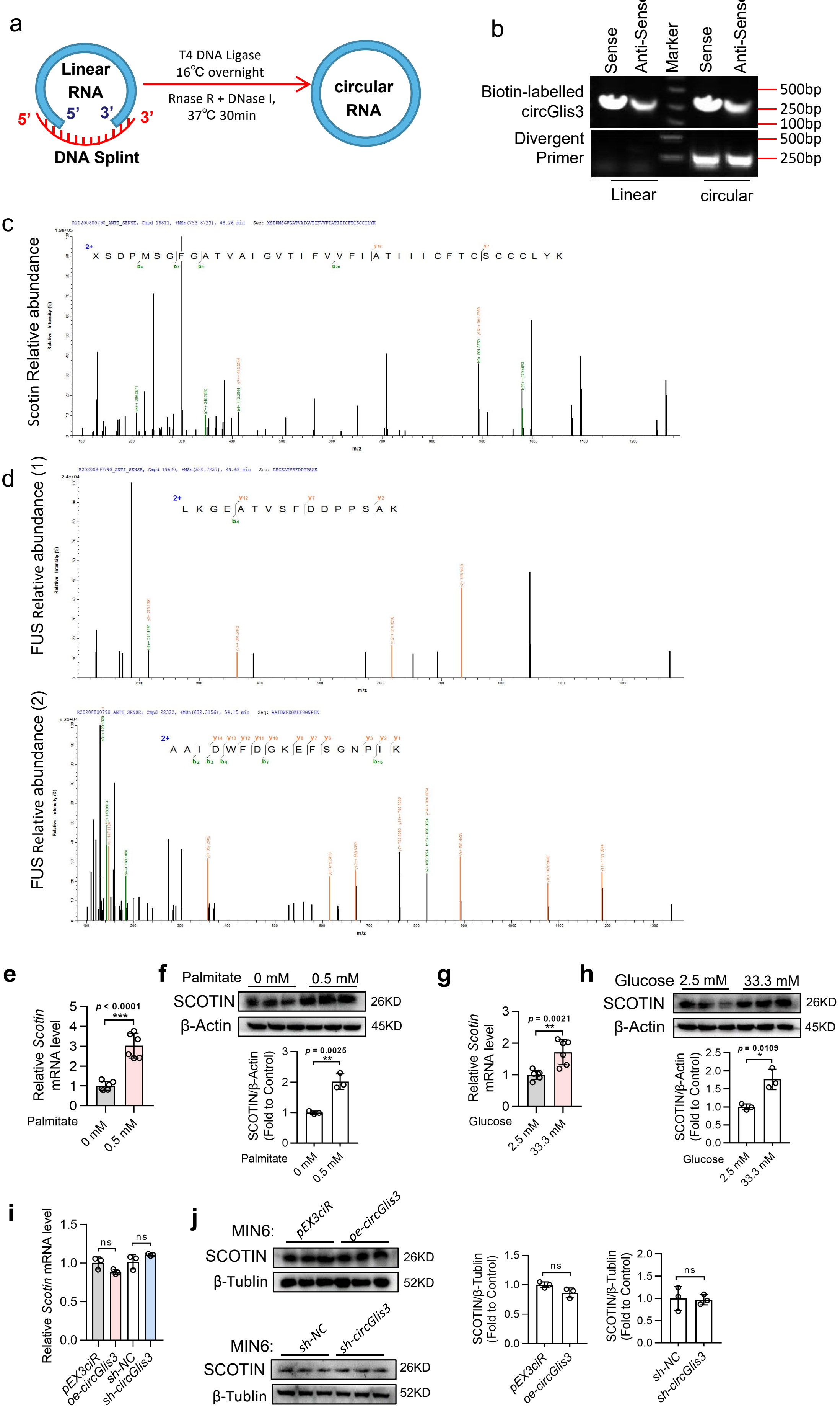

Supplementary Fig. 6 circGlis3 prevents β-cell apoptosis by directly binding to SCOTIN, Related to Fig. 6.

a. Schematic illustrating cyclization of linear RNA generated *in vitro*. **b.** PCR analysis of linear and cyclized circGlis3 RNAs with divergent primers. **c-d.** Mass spectrometry identified SCOTIN and FUS, which were pulled down from MIN6 cells lysate by biotin-labelled circGlis3. **e-f.** RT-PCR analysis and Western Blotting showing *Scotin* mRNA and protein expression in palmitate-stimulated MIN6 cells (n = 6 biological replicates). **g-h.** RT-PCR analysis and Western Blotting showing *Scotin* mRNA and protein expression in glucose-stimulated MIN6 cells (n = 6 biological replicates). **i-j.** RT-PCR analysis and Western Blotting showing *Scotin* mRNA and protein expression in MIN6 cells with circGlis3 overexpression and knockdown (n = 3 biological replicates). **e-j** For bar graphs, data represents mean ± SEM. **e-h, j** Unpaired two-tailed Student's t-test. **i** One-way ANOVA with Tukey's post-test. $^{*}p < 0.05$, $^{**}p < 0.01$, $^{***}p < 0.001$, $^{****}p < 0.0001$. Source data are provided as a Source data file.

Supplementary Fig. 7

a

Accession	G3UXT7
Description	RNA-binding protein FUS
ΣCoverage	40.00
Coverage Sense	34.62
Coverage Anti-sense	5.38

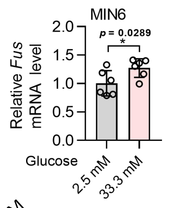

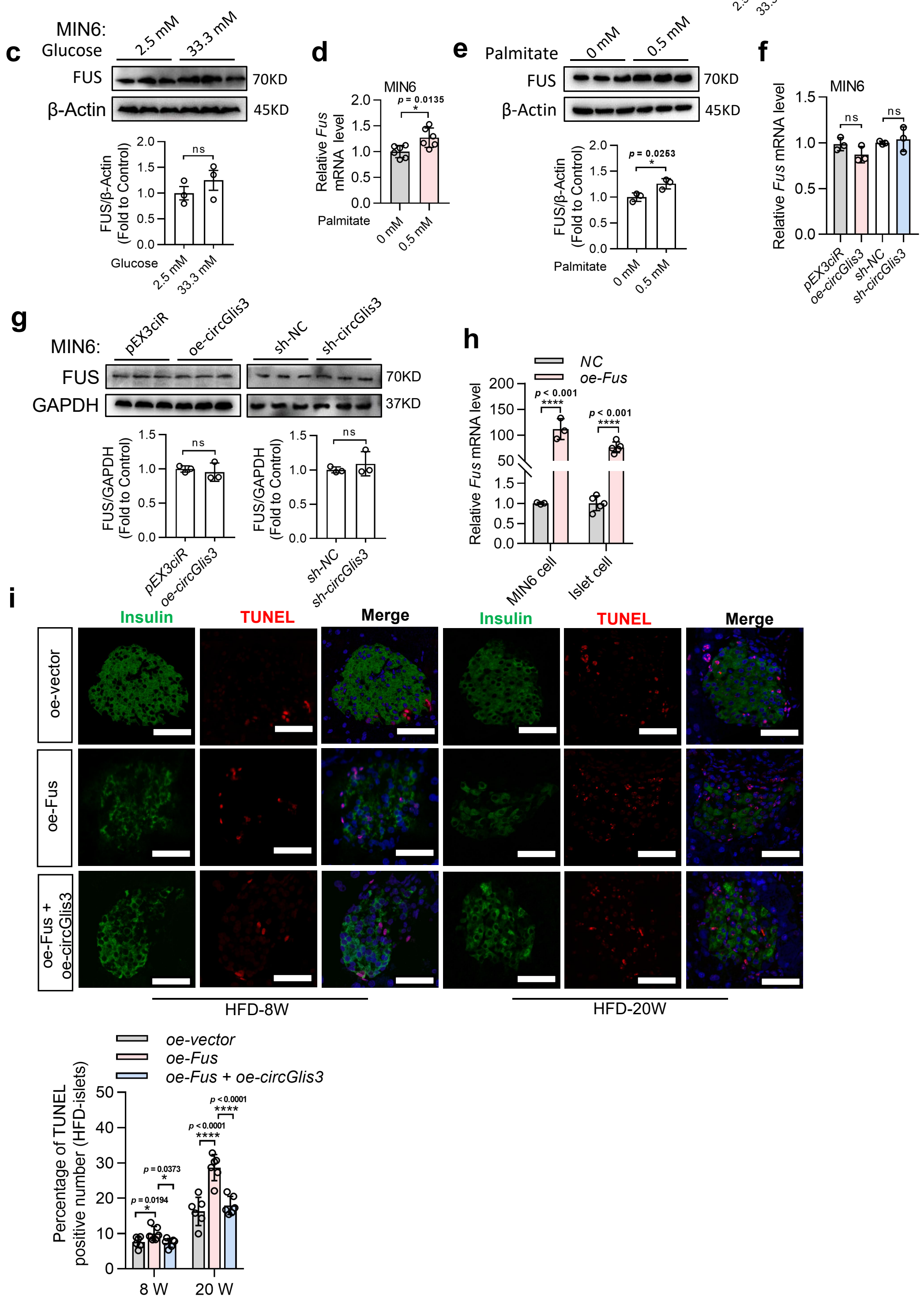

Supplementary Fig. 7 FUS sequestrates circGlis3 to reduce its abundance in diabetes by assembling cytoplasmic SG, Related to Fig. 7.

a. Relevant information of the mass spectrometry assay revealing the RNA binding protein FUS. **b-c.** RT-PCR analysis and Western Blotting showing *Fus* mRNA and protein expression in glucose-stimulated MIN6 cells (n = 6 biological replicates). **d-e.** RT-PCR analysis and Western Blotting showing *Fus* mRNA and protein expression in palmitate-stimulated MIN6 cells (n = 6 biological replicates). **f-g.** RT-PCR analysis and Western Blotting showing *Fus* mRNA and protein expression in MIN6 cells with circGlis3 overexpression and knockdown (n = 3 biological replicates). **h.** Expression efficiency of *Fus mRNA* in *oe-Fus*-treated MIN6 cells (n = 3 biological replicates) and islets (n = 5 biological subjects). **g.** TUNEL assays in pancreatic sections of the oe-circGlis3 or/and *oe-Fus*-treated mice (Red represents positive TUNEL cells, Green represents Insulin; Scale bars represent 100 μm). **b-i** For bar graphs, data represents mean ± SEM. **b-e, g** Unpaired two-tailed Student's t-test. **f** One-way ANOVA with Tukey's post-test. **h, i** Two-way ANOVA with Bonferroni's post-test. $^{*}p < 0.05$, $^{**}p < 0.01$, $^{***}p < 0.001$, $^{****}p < 0.0001$. Source data are provided as a Source data file.

Supplementary Table 1 Oligonucleotides sequences related to Figures 1-7 and Figures S1-7

The table includes qRT-PCR and RT-PCR primers, shRNA sequences, Biotin labelled probes, primers for in vitro transcription, DNA splints for in vitro cyclization, FISH probes, WT and MUT pMIR-REPORT primers, and miRNA mimics and inhibitors.

Primers used for structural identification of *circGlis3*:

Primer name		From 5’ to 3’
mmu-circGlis3	Convergent F：	AGTGTACGGTCCGTTATTTTGCAAG
	Convergent R：	GAAAGCCCTGTAGCTCTGAGACCAC
mmu-circGlis3	Divergent F：	GTCCGTTATTTTGCAAGAGTCTC
	Divergent R：	CGTACACTTGTTGGGCTTCTC
mmu-Glis3	Forward：	TGTGGCATGAATCTCCACCG
	Reverse：	AGGTGAAGACTGTTAGCAAGACT
mmu-β actin	Forward：	GGCTGTATTCCCCTCCATCG
	Reverse：	CCAGTTGGTAACAATGCCATGT
hsa-circGlis3	Convergent F：	CAAGTGTACGGTCCCTTATTTCG
	Convergent R：	CTGGGACCACTCCTGCTTCATGCTT
hsa-β actin	Forward：	CATGTACGTTGCTATCCAGGC
	Reverse：	CTCCTTAATGTCACGCACGAT

FISH probe sequence:

Probe name	From 5’ to 3’
circGlis3	GGCAT +AACGGACCGTACACT + TA
miR-124-3p	GGCAT + TCACCGCGTGCCT + TA

Overexpression vector construction of CDS sequence (Homologous recombination):

Primer name		From 5' to 3'
Qki	Forward:	gacctccatagaagacaccgATGGTCGGGGAAATGGAAAC
	Reverse:	taacgttagggggggggggagTCAATGGGCTGAAATATCAGGC
Fus	Forward:	gacctccatagaagacaccgATGGCTTCAAACGACTATACCCA
	Reverse:	taacgttagggggggggggagCTAATATGGCCTCTCCCTGCG
Scotin	Forward:	gacctccatagaagacaccgATGGCTGCGCCGGCGCCC
	Reverse:	taacgttagggggggggggagTCAGGGAATTGTCTTTAGGGAATC
NeuroD 1	Forward:	gacctccatagaagacaccgATGACCAAATCATACAGCGAGAGC
	Reverse:	taacgttagggggggggggagCTAATCGTGAAAGATGGCATTAAGC
Creb1	Forward:	gacctccatagaagacaccgATGCCAGCAGCTCATGCAA
	Reverse:	taacgttagggggggggggagTTAATCTGATTTGTGGCAGTAAAGG
pHAGE-vector	Forward:	CTCCCCCCCCCTAACGT
	Reverse:	CGGTGTCTTCTATGGAGGTC

pLVX-sh-RNA vector construction:

Primer name		From 5' to 3'
Qki-sh1	Forward:	GATCCCGAAGAAATTAGCAGAGTATTCAAGAGATACTCTGCTAATTTCTTCGTTTTTTG
	Reverse:	AATTCAAAAAACGAAGAAATTAGCAGAGTATTCAAGAGATACTCTGCTAATTTCTTCGG
Qki-sh2	Forward:	GATCCGCATCTAAATGAAGACTTATTCAAGAGATAAGTCTTCATTTAGATGCTTTTTTG
	Reverse:	AATTCAAAAAAGCATCTAAATGAAGACTTATTCAAGAGATAAGTCTTCATTTAGATGCG
Qki-sh3	Forward:	GATCCCCTTGAGTACCCTATTGAATTCAAGAGATTCAATAGGGTACTCAAGGTTTTTTG
	Reverse:	AATTCAAAAAACCTTGAGTACCCTATTGAATTCAAGAGATTCAATAGGGTACTCAAGGG

Fus-sh1	Forward:	GATCCGGAGGTGGTTACAACCGAATTCAAGAGATTCGGTTGTAACCACCTCCTTTTTTG
	Reverse:	AATTCAAAAAAGGAGGTGGTTACAACCGAATTCAAGAGATTCGGTTGTAACCACCTCCG
Fus-sh2	Forward:	GATCCCGACTGGTTTGATGGTAAATTCAAGAGATTTACCATCAAACCAGTCGTTTTTTG
	Reverse:	AATTCAAAAAACGACTGGTTTGATGGTAAATTCAAGAGATTTACCATCAAACCAGTCGG
Fus-sh3	Forward:	GATCCGACTATACCCAACAAGCAATTCAAGAGATTGCTTGTTGGGTATAGTCTTTTTTG
	Reverse:	AATTCAAAAAAGACTATACCCAACAAGCAATTCAAGAGATTGCTTGTTGGGTATAGTCG
Scotin-sh1	Forward:	GATCCGGCCCTTTGGTGAAGACAATTCAAGAGATTGTCTTCACCAAAGGGCCTTTTTTG
	Reverse:	AATTCAAAAAAGGCCCTTTGGTGAAGACAATTCAAGAGATTGTCTTCACCAAAGGGCCG
Scotin-sh2	Forward:	GATCCGTTCCTGTTCCAACCAATATTCAAGAGATATTGGTTGGAACAGGAACTTTTTTG
	Reverse:	AATTCAAAAAAGTTCCTGTTCCAACCAATATTCAAGAGATATTGGTTGGAACAGGAACG
Scotin-sh3	Forward:	GATCCCCTGCTGCTGTCTGTATAATTCAAGAGATTATACAGACAGCAGCAGGTTTTTTG
	Reverse:	AATTCAAAAAACCTGCTGCTGTCTGTATAATTCAAGAGATTATACAGACAGCAGCAGGG

Primers used for QKI-RIP qRT-PCR analysis:

Primer name		From 5' to 3'
Glis3 intron a	Forward:	TTTTCAGGGTTGGTGTGTGTGCGAT
	Reverse:	CATCGCAAACTCCATTACCTCACAA
Glis3 intron b	Forward:	CCGAGACCCTGTTTCTTCTTTTTTC
	Reverse:	AATGGGAGAGTGACAGCGCTTGCC
Glis3 intron c	Forward:	CCCAAGTAGATTTTAATTGAACACT
	Reverse:	AGTATTTTTTTATACAAAAGGGGGG
Glis3 intron d	Forward:	ACCCTCAGAGTTTAGGTGACAAG
	Reverse:	CCTTCTGGTCCATATTGGTAACA
Glis3 intron e	Forward:	TGTGAAACAAGAAGGAAATCATGCA
	Reverse:	CATCCCTGTCCTGCATTCCAAAAAG

Glis3 intron f	Forward：	TCCTTTCTTTCACTTGCACATATGT
	Reverse：	AACTCCCAAAGTCTGGATCTCTACA
Glis3 intron g	Forward：	GTGCCAGTTGTGAGGTAATGGAGTT
	Reverse：	GAGCCCAGATAAAGGAGCAGAAAGG

Primers used for *in vitro*transcription of QKI pulldown assay:

Primer name		From 5’ to 3’
(sense)	Forward：	GATCACTAATACGACTCACTATAGGGCCGAGACC CTGTTTCTTCTTTTTTC
	Reverse：	AATGGGAGAGTGACAGCGCTTGCC
QRE 3 (sense)	Forward：	GATCACTAATACGACTCACTATAGGGACCCTCAGA GTTTAGGTGACAAG
	Reverse：	CCTTCTGGTCCATATTGGTAACA
QRE 4 (sense)	Forward：	GATCACTAATACGACTCACTATAGGGGTGCCAGTT GTGAGGTAATGGAGTT
	Reverse：	GAGCCCAGATAAAGGAGCAGAAAGG

Primers used for *in vitro*transcription and *in vitro*cyclization of *circGlis3*:

Primer name		From 5’ to 3’
mmu-circGlis3 (sense)	Forward：	GATCACTAATACGACTCACTATAGTCCGTTATTT TGCAAGAGTC
	Reverse：	CGTACACTTGTTGGGCTTCTCC
mmu-circGlis3 (anti-sense)	Forward：	GATCACTAATACGACTCACTATAGGGCGTACAC TTGTTGGGCTTCTCC
	Reverse：	GTCCGTTATTTTGCAAGAGTCTC
circGlis3-sense splint		GACACCAGAGACTCTTGCAAAATAACGGACCGTA CACTTGTTGGGCTTCTCCCCAGAGT
circGlis3-anti-sense splint		ACTCTGGGGAGAAGCCCAACAAGTGTACGGTCCG TTATTTTGCAAGAGTCTCTGGTGTC

***mmu-miR-124-3p mimics* and *inhibitors*:**

Primer name	From 5’ to 3’
Mimics Negative Control	UUCUCCGAACGUGUCACGUTT
mmu-miR-124-3p mimics	UAAGGCACGCGGUGAAUGCCCAUUCACCGCGUG CCUUAUU
Inhibitor Negative Control	ACGUGACACGUUCGGAGAATT
mmu-miR-124-3p inhibitor	GGCAUUCACCGCGUGCCUUA

Primers used forpri-mmu-miR-124-3p and pre-mmu-miR-124-3p: Primers used forLuciferase assays:

Primer name		From 5' to 3'
pri-mmu-miR-124-3p	Forward:	GAATGAACCCATCCTGTGCG
	Reverse:	GAGTGCCATCTCTTAGACCGTTT
	Size:	198 bp
pre-mmu-miR-124-3p	Forward:	GTTCACAGCGGACCTTGATTA
	Reverse:	GCTCCGCTCTTGGCATTC
	Size:	68 bp

Primers used for Luciferase assays:

Primer name		From 5' to 3'
circGlis3-miR-298-WT	Forward:	TCGAGGTTCCCCGC<u>CAGTGCCTTGGACCTGC CCTCTGCT</u>CTGCCTCTCCCTCTTCCT
circGlis3-miR-298-WT	Reverse:	CTAGAGGAAGAGGGAGAGGCAGAGCAGAGG GCAGGTCCAAGGCACTGGCGGGGAACC
circGlis3-miR-298-MUT	Forward:	TCGAGGTTCCCCGC<u>CAGTGCCTGCTATTGGT TTGAGATT</u>CTGCCTCTCCCTCTTCCT
circGlis3-miR-298- MUT	Reverse:	CTAGAGGAAGAGGGAGAGGCAGAATCTCAAA CCAATAGCAGGCACTGGCGGGGAACC
circGlis3-miR-124-WT	Forward:	TCGAGAGAGCGCCTGGA<u>GGAGTTCCCCGCC AGTGCCTTG</u>GACCTGCCCTCTGCTT
circGlis3-miR-124-WT	Reverse:	CTAGAAGCAGAGGGCAGGTCCAAGGCACTG GCGGGGAACTCCTCCAGGCGCTCTC
circGlis3-miR-124- MUT	Forward:	TCGAGAGAGCGCCTGGA<u>GGACGGAAAATTTG TGAAACCG</u>GACCTGCCCTCTGCTT
circGlis3-miR-124- MUT	Reverse:	CTAGAAGCAGAGGGCAGGTCCGGTTTCACAA ATTTTCCGTCCTCCAGGCGCTCTC
circGlis3-miR-3113-WT	Forward:	TCGAGGCTGGATCGACTGCAGCGCCCTGTAC GACCAGCAGGAGGAGCTCGTGCGGCACT
circGlis3-miR-3113-WT	Reverse:	CTAGAGTGCCGCACGAGCTCCTCCTGCTGGT CGTACAGGGCGCTGCAGTCGATCCAGCC
circGlis3-miR-3113- MUT	Forward:	TCGAGGCTGGATCGACTGCAGCGCCCTGTAC GACAAAAGAAAGGGAGCTCGTGCGGCACT
circGlis3-miR-3113- MUT	Reverse:	CTAGAGTGCCGCACGAGCTCCCTTTCTTTTG TCGTACAGGGCGCTGCAGTCGATCCAGC
circGlis3-miR-3104-WT	Forward:	TCGAGTTCCTCCCGGGACATCCATGTCCAGC AATAGTGTCTCTAACTCATTACCATCCT

circGlis3-miR-3104-WT	Reverse:	CTAGAGGATGGTAATGAGTTAGAGACACTATT GCTGGACATGGATGTCCCGGGAGGAAC
circGlis3-miR-3104- MUT	Forward:	TCGAGTTCCTCCCGGGACATCCATGTCCGAA GGTAGTGGGGGGGGCTCATTACCATCCT
circGlis3-miR-3104- MUT	Reverse:	CTAGAGGATGGTAATGAGCCCCCCCCACTAC CTTCGGACATGGATGTCCCGGGAGGAAC
miR-124-NeuroD1-WT	Forward:	TCGAGACGGAAAGTCAGTCGGCCTCAAGCAT GAAGCAGGAGTGGTCCCAGGGCTACAGT
miR-124-NeuroD1-WT	Reverse:	CTAGACTGTAGCCCTGGGACCACTCCTGCTT CATGCTTGAGGCCGACTGACTTTCCGTC
miR-124-NeuroD1- MUT	Forward:	TCGAGACGGAAAGTCAGTCGGCCTCAAGCAT tAGTCGAACTGTGTCCCAGGGCTACAGT
miR-124-NeuroD1- MUT	Reverse:	CTAGACTGTAGCCCTGGGACACAGTTCGACT AATGCTTGAGGCCGACTGACTTTCCGTC
miR-124-Creb1-WT	Forward:	TCGAGATCTAGGGGATCTCCTTAGCCTTCCT CCCGGGACATCCATGTCCAGCAATAGTT
miR-124-Creb1-WT	Reverse:	CTAGAACTATTGCTGGACATGGATGTCCCGG GAGGAAGGCTAAGGAGATCCCCTAGATC
miR-124-Creb1- MUT	Forward:	TCGAGATCTAGGGGATCTCCTTAGCCTTCCT CCCGGGACATCCAGGGCCCTAGGCTATT
miR-124-Creb1- MUT	Reverse:	CTAGAATCGCCTAGGGCCCTGGATGTCCCGG GAGGAAGGCTAAGGAGATCCCCTAGATC

Other primers used for qRT-PCR assay:

		From 5' to 3'
mmu-Qki	Forward:	ATGGTCGGGGAAATGGAAACG
	Reverse:	TTAGTTGCCGGTGGCGGCTCGGTCT
mmu-Glis3	Forward:	AGGCAGGACTGGATAGTCAGA
	Reverse:	AGGCATCCTTAAAGTGAGACTCT
mmu-Creb1	Forward:	CTGAGAGCTGGTATGTCAGGA
	Reverse:	TGAGTGCTGGAGTAAAACAGTCA
mmu-NeuroD1	Forward:	ATGACCAAATCATACAGCGAGAG
	Reverse:	TCTGCCTCGTGTTCCTCGT
mmu-Scotin	Forward:	CCCTCTCTGTGGACCCTATTG
	Reverse:	GGGATCGAATTGTCTTCACCAA
mmu-Fus	Forward:	GCTTCAAACGACTATACCCAACA
	Reverse:	GGCCATAACCACTGTAACTCTGT

mmu-Esrp1	Forward：	CAAGCTGGGTTCGGATGAGAA
	Reverse：	AGGTTTTCGGCGTCTATTTTAGT
mmu-Esrp2	Forward：	CCAGATCCCGCAGTAGACTC
	Reverse：	AGGATTAAGTCGGTCTCGTCC
mmu-Mbl	Forward：	CTTCTGCTTCCATTACTCCCTG
	Reverse：	GAGGCCCCAATTTCCCTGG
mmu-Nova1	Forward：	TCCAGGTACTACTGAGAGGGT
	Reverse：	TTGGGGCATTTCTCGGATTTT
mmu-Nova2	Forward：	CGGCTCAATCATCGGCAAAG
	Reverse：	GCATACCCGTTCTGTAGTTCCTG
mmu-Rbm6	Forward：	TCTCGATCTGCTAACAGAACAGG
	Reverse：	CTCCCCTCCTTGATAGTCACC
mmu-Msi1	Forward：	ACAGGTCAGTGGCTCAGATTC
	Reverse：	CCAGGTACAGTCGTTCTGCC
mmu-Ifit	Forward：	CTGAGATGTCACTTCACATGGAA
	Reverse：	GTGCATCCCCAATGGGTTCT
mmu-srsf1	Forward：	AACAACGACTGCCGCATCTAC
	Reverse：	TCGATGTCCTTGGTTCGGATA
mmu-srsf2	Forward：	CGCGCTCCAGATCAACCTC
	Reverse：	CTTGGACTCTCGCTTCGACAC
mmu-Mbnl2	Forward：	TGATCGACACAAACGACAACA
	Reverse：	GAGAGGTCGCTTCAGTGCT
mmu-Ptbp1	Forward：	CCTCTCCGTATGCAGGAGC
	Reverse：	CCGTAGACGCCGAAGAGAATAA
mmu-Ptbp2	Forward：	ATGGACGGAATTGTCACTGAGG
	Reverse：	TGCCACTCATATTAGAGTTGGGG
mmu-Cbx7	Forward：	TGCGGAAGGGCAAAGTTGAAT
	Reverse：	ACAAGGCGAGGGTCCAAGA
mmu-Ins1	Forward：	CACTTCCTACCCCTGCTGG
	Reverse：	ACCACAAAGATGCTGTTTGACA
mmu-Ins2	Forward：	GCTTCTTCTACACACCCATGTC
	Reverse：	AGCACTGATCTACAATGCCAC
mmu-Pdx1	Forward：	CCCCAGTTTACAAGCTCGCT
	Reverse：	CTCGGTTCCATTCGGGAAAG

mmu-Nkx6.1	Forward:	CTGCACAGTATGGCCGAGATG
	Reverse:	CCGGGTTATGTGAGCCCAA
mmu-Mafa	Forward:	AGGAGGAGGTCATCCGACTG
	Reverse:	CTTCTCGCTCTCCAGAATGTG
mmu-β-Actin	Forward:	GGCTGTATTCCCCTCCATCG
	Reverse:	CCAGTTGGTAACAATGCCATGT
mmu-Gapdh	Forward:	AGGTCGGTGTGAACGGATTTG
	Reverse:	TGTAGACCATGTAGTTGAGGTCA
hsa-Qki	Forward:	AAGCCCACCCCAGATTACCT
	Reverse:	ACTCTGCTAATTTCTTCGTCCAG
hsa-circGlis3	Forward:	CAAGTGTACGGTCCCTTATTTCG
	Reverse:	CTGGGACCACTCCTGCTTCATGCTT
hsa-β-Actin	Forward:	CATGTACGTTGCTATCCAGGC
	Reverse:	CTCCTTAATGTCACGCACGAT

Primers used for qRT-PCR in Figure S3G-H:

Primer name		From 5' to 3'
Arx	Forward:	GGCCGGAGTGCAAGAGTAAAT
	Reverse:	TGCATGGCTTTTTCCTGGTCA
bHLHa15	Forward:	GCTGACCGCCACCATACTTAC
	Reverse:	TGTGTAGAGTAGCGTTGCAGG
c-Maf	Forward:	GGAGACCGACCGCATCATC
	Reverse:	TCATCCAGTAGTAGTCTTCCAGG
Creb1	Forward:	AGCAGCTCATGCAACATCATC
	Reverse:	AGTCCTTACAGGAAGACTGAACT
Etv5	Forward:	TCAGTCTGATAACTTGGTGCTTC
	Reverse:	GGCTTCCTATCGTAGGCACAA
Foxa2	Forward:	CCCTACGCCAACATGAACTCG
	Reverse:	GTTCTGCCGGTAGAAAGGGA
FoxO1	Forward:	CCCAGGCCGGAGTTTAACC
	Reverse:	GTTGCTCATAAAGTCGGTGCT
FOXP3	Forward:	CCCATCCCCAGGAGTCTTG
	Reverse:	ACCATGACTAGGGGCACTGTA
GATA3	Forward:	CTCGGCCATTCGTACATGGAA
	Reverse:	GGATACCTCTGCACCGTAGC
GATA4	Forward:	CCCTACCCAGCCTACATGG
	Reverse:	ACATATCGAGATTGGGGTGTCT

GATA6	Forward：	CCCTACCCAGCCTACATGG
	Reverse：	ACATATCGAGATTGGGGTGTCT
Grg3	Forward：	GACAAAGCCCGTGGTCTGAA
	Reverse：	CAGGGCCGAGGCCATTATAC
ISl1	Forward：	ATGATGGTGGTTTACAGGCTAAC
	Reverse：	TCGATGCTACTTCACTGCCAG
Hmgb1	Forward：	GGCGAGCATCCTGGCTTATC
	Reverse：	GGCTGCTTGTCATCTGCTG
Hnf1a	Forward：	GACCTGACCGAGTTGCCTAAT
	Reverse：	CCGGCTCTTTCAGAATGGGT
Hnf1b	Forward：	CCCCTCACCATCAGCCAAG
	Reverse：	GGTTCTGAGATTGCTGGGGATT
Hnf4a	Forward：	CACGCGGAGGTCAAGCTAC
	Reverse：	CCCAGAGATGGGAGAGGTGAT
Hnf6	Forward：	GGCAACGTGAGCGGTAGTTT
	Reverse：	TTGCTGGGAGTTGTGAATGCT
MafA	Forward：	AGGAGGAGGTCATCCGACTG
	Reverse：	CTTCTCGCTCTCCAGAATGTG
MafB	Forward：	TTCGACCTTCTCAAGTTCGACG
	Reverse：	TCGAGATGGGTCTTCGGTTCA
Mnx1	Forward：	TCTATGGACACCCGGTCTACA
	Reverse：	CCCCAAGAGGTTCGACTGC
NeuroD1	Forward：	ATGACCAAATCATACAGCGAGAG
	Reverse：	TCTGCCTCGTGTTCCTCGT
Ngn3	Forward：	AGTGCTCAGTTCCAATTCCAC
	Reverse：	CGGCTTCTTCGCTTTTTGCTG
Nkx2.2	Forward：	AAGCATTTCAAAACCGACGGA
	Reverse：	CCTCAAATCCACAGATGACCAGA
Nkx6.1	Forward：	CTGCACAGTATGGCCGAGATG
	Reverse：	CCGGGTTATGTGAGCCCAA
Pax4	Forward：	AGGGGGACTCTTTGTGAATGG
	Reverse：	ACCTGTGCGGTAGTAGCGT
Pax6	Forward：	TACCAGTGTCTACCAGCCAAT
	Reverse：	TGCACGAGTATGAGGAGGTCT
Pbx1	Forward：	CAGCGGGTTCTTCCAGTTCTT
	Reverse：	CGAGTCCGTCACTGTATCCTC

Ptf1a	Forward:	TCCCATCCCCTTACTTTGATGA
	Reverse:	GTAGCAGTATTCGTGTAGCTGG
Prep1	Forward:	GGTGGTCACGGAGTTAAAAACA
	Reverse:	TCGGCATCCATTGGGGTCT
Pparg	Forward:	TCGCTGATGCACTGCCTATG
	Reverse:	GAGAGGTCCACAGAGCTGATT
Rfx6	Forward:	GCTTGCTGGTCTACCCTGAG
	Reverse:	TGCCGTTGTTTAACTGCATTTC
Slc2a2	Forward:	TCAGAAGACAAGATCACCGGA
	Reverse:	GCTGGTGTGACTGTAAGTGGG
Smad4	Forward:	ACACCAACAAGTAACGATGCC
	Reverse:	GCAAAGGTTTCACTTTCCCCA
Sox4	Forward:	CGGCTGCATCGTTCTCTCC
	Reverse:	GGTAGACGTGCTTCACTTTCTTG
Sox9	Forward:	GAGCCGGATCTGAAGAGGGA
	Reverse:	GCTTGACGTGTGGCTTGTTC
Stat1	Forward:	TCACAGTGGTTCGAGCTTCAG
	Reverse:	GCAAACGAGACATCATAGGCA
Stat3	Forward:	CAATACCATTGACCTGCCGAT
	Reverse:	GAGCGACTCAAACTGCCCT
Stat4	Forward:	TGGCAACAATTCTGCTTCAAAAC
	Reverse:	GAGGTCCCTGGATAGGCATGT
Tbp	Forward:	AGAACAATCCAGACTAGCAGCA
	Reverse:	GGGAACTTCACATCACAGCTC
TCF7L2	Forward:	AACGAACACAGCGAATGTTTCC
	Reverse:	CTCGGCATTTCTTAGGAGCG
TGF-beta1	Forward:	CTCCCGTGGCTTCTAGTGC
	Reverse:	GCCTTAGTTTGGACAGGATCTG
TNF	Forward:	CCCTCACACTCAGATCATCTTCT
	Reverse:	GCTACGACGTGGGCTACAG
TP53	Forward:	GCGTAAACGCTTCGAGATGTT
	Reverse:	TTTTTATGGCGGGAAGTAGACTG
Ppia	Forward:	GAGCTGTTTGCAGACAAAGTTC
	Reverse:	CCCTGGCACATGAATCCTGG
B2M	Forward:	TTCTGGTGCTTGTCTCACTGA
	Reverse:	CAGTATGTTCGGCTTCCCATTC

***circGlis3* sequence (5’-3‘):**

GTCCGTTATTTTGCAAGAGTCTCTGGTGTCCACGACTTTGAGTCTGACAGAGAGTCA
ATCAGCCTTGAGTGTGAAGCAAGAGTGGTCTCAGAGCTACAGGGCTTTCCCTTCACT
TTCATCCAGCCACAGTTCCCAGAATGGCACGGACCTAGGGGACCTTCTTAGCTTGCC
TCCAGGCACGCCAGTGTCTGGCAACAGCGTCTCCAACTCGTTGCCACCCTACCTTTT
CGGCATGGAAAATAGCCACTCTCCTTACCCTAGCCCTCGGCACTCAGCAACCAGGG
CCCACTCCACCCGCTCTAAGAAGAGAGCATTGTCCTTGTCGCCACTGTCAGATGGCA
TCGGGATCGACTTCAACACTATCATCCGTACTTCACCCACATCCTTGGTCGCCTACAT
CAACGGACCAAGAGCCTCCCCAGCCAACCTGTCCCCACAGTCAGAGGTCTATGGGC
ATTTCCTGGGTGTTCGTGGCAGCTGCATCCCCCAGTCTTGTGCAGTGGCCAGCGGG
CAGAAAGGCATATTGGTTGCCAGTGGAGGGCATACGCTGCCGGGCTATGGAGAGGA
CGGTACACTGGAGTATGAACGCATGCAGCAGCTTGAGCATGGTGGCCTGCAACCCG
GACCTGTAAACAACATGGTGTTGCAGCCTGGCCTACCGGGCCAGGATGGCCAGACA
GCCAACATGCTCAAAACAGAGCGCCTGGAGGAGTTCCCCGCCAGTGCCTTGGACCT
GCCCTCTGCTCTGCCTCTCCCTCTTCCTCCGCCTCAGGGTCCCCCACCCCCATACCA
TGCCCATCCACACCTTCATCACCCAGAGCTCCTGCCTCACACCCAATCGCTGTCCCT
GGCCCAGACTGGCCTGGAAGAGGATGGGGAGATGGAAGACTCAGGGGGGAAGCAC
TGCTGCCGTTGGATAGACTGCAGTGCCCTTTATGACCAGCAGGAGGAACTGGTGAG
GCACATCGAGAAGGTCCACATAGACCAACGCAAGGGGGAAGACTTCACGTGCTTCT
GGACTGGCTGCCCTAGAAGATACAAGCCTTTCAACGCACGGTATAAACTGCTGATCC
ACATGAGGGTCCACTCTGGGGAGAAGCCCAACAAGTGTACG

Supplementary Table 2

Supplementary Table 2 Key Resources Table

RESOURCE	SOURCE	IDENTIFIER
**Antibodies**		
Glis3 (WB 1:1000)	Proteintech	Cat# 12678-1-AP
β-actin (WB 1:1000)	CST	Cat# 3700
β-Tublin (WB 1:1000)	CST	Cat# 2148
GAPDH (WB 1:1000)	CST	Cat# 5174
Creb1 (WB 1:1000)	Proteintech	Cat# 67927-1-Ig
Bcl-2 (WB 1:1000)	Proteintech	Cat# 26593-1-AP
Bax (WB 1:1000)	Proteintech	Cat# 50599-2-Ig
Caspase 3 (WB 1:1000)	Proteintech	Cat# 19677-1-AP
Qki (WB 1:1000)	CST	Cat# 86397
NeuroD1 (WB 1:1000)	Abcam	Cat# ab109224
cleaved-Caspase 3 (WB 1:1000)	CST	Cat# 9664
FUS (WB 1:1000; IF 1:100)	CST	Cat# 67804
Nkx6.1 (WB 1:1000)	CST	Cat# 54551
Mafa (WB 1:1000)	CST	Cat# 79737
Pdx1 (WB 1:1000)	CST	Cat# 5679
Scotin (WB 1:1000; IF 1:100)	Sigma	Cat# HPA042295
Insulin (Rabbit, IF 1:200)	CST	Cat# 3014
Insulin (Mouse, IF 1:200)	CST	Cat# 8138
Goat anti-rabbit IgG H&L (Alexa Fluor® 488) (IF 1:500)	Abcam	Cat# ab150077
Goat anti-mouse IgG H&L (Alexa Fluor® 488) (IF 1:500)	Abcam	Cat# ab150113
Goat anti-mouse IgG H&L (Alexa Fluor® 488) (IF 1:500)	Abcam	Cat# ab150115
Donkey anti- rabbit IgG H&L (Alexa Fluor® 674) (IF 1:500)	Abcam	Cat# ab150075

**Chemicals**		
Palmitate	Sigma	Cat# P9767
Gemcitabine	Solarbio	Cat# 95058-81-4
Ketoprofen	Solarbio	Cat# 22071-15-4
Isoflurane	RWD	Cat# R510-22-16
EMETINE	Selleck	Cat#S3233

Critical Commercial Reagent

DMEM	Gibco	Cat# 12800082
RPMI-1640	Gibco	Cat# 61870044
DMEM, no Glucose	Gibco	Cat# 11966025
Fetal Bovine Serum	Gibco	Cat# 10371029
CMRL-1066 medium	Corning	Cat# 15-110-CV
Human Serum Albumin	Baxter	Cat# S20160018
TRIzol	Solarbio	Cat# R1100
PARIS Kit	Invitrogen	Cat# AM1921
BCA Protein Assay Kit	Beyotime	Cat# P0012
Annexin V Apoptosis Detection Kit	Beyotime	Cat# C1062M
TUNEL Apoptosis Assay Kit (Cy3)	Beyotime	Cat# C1089
TUNEL Apoptosis Assay Kit (FITC)	Beyotime	Cat# C1088
Magnetic RNA-Protein Pull-Down Kit	Thermo Fisher Scientific	Cat# 20164
TranscriptAid T7 High Yield Transcription Kit	Thermo Fisher Scientific	Cat# K0441
RNA 3' End Biotinylation Kit	Thermo Fisher Scientific	Cat# 20160
Magna RIP RNA-Binding Protein Immunoprecipitation Kit	Millipore	Cat# 17-700
T4 DNA ligase	Takara	Cat# D2011A
RIPA buffer	Beyotime	Cat# P0013B
Dual-Luciferase Reporter Assay	Beyotime	Cat# RG017
Plasma/serum RNA Purification Kit	Sigma	Cat# RNB500
ChamQ qPCR SYBR Green Master Mix	Vazyme	Cat# Q121-02
Phanta Max Super-Fidelity DNA Polymerase	Vazyme	Cat# P505-d1
DNA Gel Extraction Kit	Vazyme	Cat# DC301-01

Animal Models and Cell Lines

C57BL/6J	Model Animal Research Center of Nanjing University	N/A
Lepr$^{db/db}$mice	Model Animal Research Center of Nanjing University	N/A
Lep$^{ob/ob}$mice	Model Animal Research Center of Nanjing University	N/A

Oligonucleotides

FISH probe sequence for *circGlis3* and *miR-124-3p*	Gene pharma, Shanghai, see Table S1	N/A
mmu-miR-124-3p mimics and *inhibitors*	Gene pharma, Shanghai, see Table S1	N/A
shRNA and siRNA targeting sequences	see Table S1	N/A
qRT-PCR and RT-PCR primers	see Table S1	N/A
Primers for in vitro transcription	see Table S1	N/A
DNA splints for in vitro cyclization	see Table S1	N/A
RIP primers	see Table S1	N/A

Recombinant DNA

pEx-3 ciR (circRNA expressing vector)		N/A
pGPU6 ciR (circRNA shRNA vector)	Gene pharma, Shanghai	N/A
pHAGE-Qki	see Table S1	N/A
pHAGE-Fus	see Table S1	N/A
pHAGE-Scotin	see Table S1	N/A
pHAGE_puro vector	see Table S1	N/A
AAV8-pAV-CMV-circGlis3-EFFS	ViGene Biosciences, Shandong	N/A
LV2N-mmu-miR-124-3p	Gene pharma, Shanghai	N/A
Lentivirus -Puro-CMV-Fus	Corues, Nanjing	N/A
Lentivirus -Puro-CMV-Scotin	Corues, Nanjing	N/A

Software and Algorithms

GraphPad Prism 7	GraphPad Software	https://www.graphpad.com/
ImageJ	NIH	https://imagej.nih.gov/ij/
SPSS 22.0	International Business, Machines Corporation	http://www.spss.com.cn/3
FlowJo 10.0	Treestar	https://www.flowjo.com/solutions/flowjo
BD Accuri C6	Accuri cytometers	N/A

ZEN 2012 Light Edition	Zeiss	N/A
Primer 5	PREMIER Biosoft International	http://www.premierbiosoft.com/primerdesign/
