# Supplementary material for: Circular RNA circGlis3 protects against islet β-cell dysfunction and apoptosis in obesity
Source: Nat Commun. 2023 Jan 21;14:351. doi: 10.1038/s41467-023-35998-z (PMC9867769; doi:10.1038/s41467-023-35998-z)
Supplement: Supplementary file 3 — Reporting Summary [file 41467_2023_35998_MOESM3_ESM.pdf]

nature portfolio

Corresponding author(s): Liang Jin

Last updated by author(s): Dec 22, 2022

Reporting Summary

Nature Portfolio wishes to improve the reproducibility of the work that we publish. This form provides structure for consistency and transparency in reporting. For further information on Nature Portfolio policies, see our Editorial Policies and the Editorial Policy Checklist.

Statistics

For all statistical analyses, confirm that the following items are present in the figure legend, table legend, main text, or Methods section.

n/a	Confirmed	
☐	☒	The exact sample size (n) for each experimental group/condition, given as a discrete number and unit of measurement
☐	☒	A statement on whether measurements were taken from distinct samples or whether the same sample was measured repeatedly
☐	☒	The statistical test(s) used AND whether they are one- or two-sided<br>*Only common tests should be described solely by name; describe more complex techniques in the Methods section.*
☒	☐	A description of all covariates tested
☒	☐	A description of any assumptions or corrections, such as tests of normality and adjustment for multiple comparisons
☐	☒	A full description of the statistical parameters including central tendency (e.g. means) or other basic estimates (e.g. regression coefficient) AND variation (e.g. standard deviation) or associated estimates of uncertainty (e.g. confidence intervals)
☐	☒	For null hypothesis testing, the test statistic (e.g. F, t, r) with confidence intervals, effect sizes, degrees of freedom and P value noted<br>*Give P values as exact values whenever suitable.*
☒	☐	For Bayesian analysis, information on the choice of priors and Markov chain Monte Carlo settings
☒	☐	For hierarchical and complex designs, identification of the appropriate level for tests and full reporting of outcomes
☒	☐	Estimates of effect sizes (e.g. Cohen's d, Pearson's r), indicating how they were calculated

Our web collection on statistics for biologists contains articles on many of the points above.

Software and code

Policy information about availability of computer code

Data collection	Carl Zeiss LSM700, Tanon 3500 system, BD FACSDiva software, BD Accuri C6 Software
Data analysis	ImageJ (v1.8.0), BD Accuri C6 Software, FlowJo 10.0, ZEN 2012, GraphPad Prism 7, SPSS 22.0

For manuscripts utilizing custom algorithms or software that are central to the research but not yet described in published literature, software must be made available to editors and reviewers. We strongly encourage code deposition in a community repository (e.g. GitHub). See the Nature Portfolio guidelines for submitting code & software for further information.

Data

Policy information about availability of data

All manuscripts must include a data availability statement. This statement should provide the following information, where applicable:

- Accession codes, unique identifiers, or web links for publicly available datasets
- A description of any restrictions on data availability
- For clinical datasets or third party data, please ensure that the statement adheres to our policy

Data availability

The data that support the findings of this study are available from the corresponding author upon reasonable request. circGlis3 was recorded in circBase database (http://circbase.org/cgi-bin/simplesearch.cgi). The RNA-seq data that support the findings of this study are available at Gene Expression Omnibus (GEO), with the accession number GSE139991 (https://www.ncbi.nlm.nih.gov/geo/query/acc.cgi?acc=GSE139991), Sequence Read Archive (SRA) database, with accession number

PRJNA835620 (https://www.ncbi.nlm.nih.gov/sra?linkname=bioproject_sra_all&from_uid=835620). The source data underlying Figs. 1a-i; 2d-e, g-k, m-r; 3a-m; 4b-s; 5b-e, g-v; 6b-c, e-m; 7a-k, n. Supplementary figs. 1a-p; 2b-h; 3a-i; 4a-j; 5a-b, d-f, k-n; 6c-j; 7b-i are provided as source data file.

Human research participants

Policy information about studies involving human research participants and Sex and Gender in Research.

Reporting on sex and gender	Only information on sex was collected for this study. Information is provided in Table 1 and source data file Supplementary figs. 1o-p., a summary is given in the 'Population characteristics' field below. Gender was not collected for this study.
Population characteristics	Participants without IGT (BMI ≤ 24); median age/range: 49 (40 - 57) years; male:female ratio: 20:9 (2.2)<br>Participants with IGT (24 < BMI ≤ 30): median age/range: 50 (38 - 62) years; male:female ratio: 43:32 (1.3)<br>Participants with IGT (BMI > 30): median age/range: 50 (42 - 59) years; male:female ratio: 9:5 (1.8)
Recruitment	Suitable samples were identified in retrospective using the laboratory information system of the hospital.
Ethics oversight	Ethics Committees of the Department Zhongda Hospital Southeast University (Nanjing, China, 2018ZDSYLL132-P01)

Note that full information on the approval of the study protocol must also be provided in the manuscript.

Field-specific reporting

Please select the one below that is the best fit for your research. If you are not sure, read the appropriate sections before making your selection.

[x] Life sciences [ ] Behavioural & social sciences [ ] Ecological, evolutionary & environmental sciences

For a reference copy of the document with all sections, see nature.com/documents/nr-reporting-summary-flat.pdf

Life sciences study design

All studies must disclose on these points even when the disclosure is negative.

Sample size	Minimum sample size per group was set at 5-6, similarly to previously published methods (e.g. doi:10.1038/s41467-022-35167-8, published online 2022 Nov 29.)
Data exclusions	No data were excluded from the analyses in this study.
Replication	Reported results considered biological replicates (in vivo n=6-9, and in vitro n=3-6). All details on biological and technical replicates are provided in the text and/or figure legends. All the experiments and data shown in this manuscript have been repeated independently at least three times with similar results as indicated in the legends.
Randomization	Allocation was random.
Blinding	Blinding was not done as most of the experiments were carried out by one or two persons and the data were collected and analyzed by the same person. It was not feasible during the course of the study to have at least 1-2 individuals for each experiment. No behavioral experiments were included in this study, no data was excluded in this study and all analyses were performed in a quantitative and objective way.

Reporting for specific materials, systems and methods

We require information from authors about some types of materials, experimental systems and methods used in many studies. Here, indicate whether each material, system or method listed is relevant to your study. If you are not sure if a list item applies to your research, read the appropriate section before selecting a response.

Materials & experimental systems

n/a	Involved in the study	
[ ]	[x]	Antibodies
[ ]	[x]	Eukaryotic cell lines
[x]	[ ]	Palaeontology and archaeology
[ ]	[x]	Animals and other organisms
[x]	[ ]	Clinical data
[x]	[ ]	Dual use research of concern

Methods

n/a	Involved in the study	
[x]	[ ]	ChIP-seq
[ ]	[x]	Flow cytometry
[x]	[ ]	MRI-based neuroimaging

Antibodies

Antibodies used	Glis3 (WB 1:1000), Proteintech, Cat# 12678-1-AP<br>!-actin (WB 1:1000), CST, Cat# 3700<br>!-Tublin (WB 1:1000), CST, Cat# 2148<br>GAPDH (WB 1:1000), CST, Cat# 5174<br>Creb1 (WB 1:1000), Proteintech, Cat# 67927-1-Ig<br>Bcl-2 (WB 1:1000), Proteintech, Cat# 26593-1-AP<br>Bax (WB 1:1000), Proteintech, Cat# 50599-2-Ig<br>Caspase 3 (WB 1:1000), Proteintech, Cat# 19677-1-AP<br>Qki (WB 1:1000), CST, Cat# 86397<br>NeuroD1 (WB 1:1000), Abcam, Cat# ab109224<br>cleaved-Caspase 3 (WB 1:1000), CST, Cat# 9664<br>FUS (WB 1:1000; IF 1:100), CST, Cat# 67840<br>Nkx6.1 (WB 1:1000), CST, Cat# 54551<br>MafA (WB 1:1000), CST, Cat# 79737<br>Pdx1 (WB 1:1000), CST, Cat# 5679<br>Scotin (WB 1:1000; IF 1:100), Sigma, Cat# HPA042295<br>Insulin (Rabbit, IF 1:200), CST, Cat# 3014<br>Insulin (Mouse, IF 1:200), CST, Cat# 8138<br>Goat anti-rabbit IgG H&L (Alexa Fluor® 488) (IF 1:500), Abcam, Cat# ab150077<br>Goat anti-mouse IgG H&L (Alexa Fluor® 488) (IF 1:500), Abcam, Cat# ab150113<br>Goat anti-mouse IgG H&L (Alexa Fluor® 488) (IF 1:500), Abcam, Cat# ab150115<br>Donkey anti- rabbit IgG H&L (Alexa Fluor® 647) (IF 1:500), Abcam, Cat# ab150075
Validation	All primary antibodies for western blot are rabbit, primary antibodies for immunofluorescence are rabbit or mouse.<br>Glis3 (WB 1:1000), Proteintech, Cat# 12678-1-AP; Species: Human, Mouse, Rat; Apply to WB, ELISA<br>!-actin (WB 1:1000), CST, Cat# 3700; Species: Human, Mouse, Rat, Hamster, Monkey, Dog; Apply to IF, IHC, WB, FCM<br>!-Tublin (WB 1:1000), CST, Cat# 2148; Species: Human, Mouse, Rat, Monkey, Zebrafish, Cow; Apply to IF, IHC,WB, FCM<br>GAPDH (WB 1:1000), CST, Cat# 5174; Species: Human, Mouse, Rat, Monkey; Apply to IF, IHC, WB<br>Creb1 (WB 1:1000), Proteintech, Cat# 67927-1-Ig; Species: Human, Mouse, Rat; Apply to IHC,WB<br>Bcl-2 (WB 1:1000), Proteintech, Cat# 26593-1-AP; Species: Human, Mouse, Rat; Apply to IF, IHC,WB, ELISA<br>Bax (WB 1:1000), Proteintech, Cat# 50599-2-Ig; Species: Human, Mouse, Rat; Apply to IP, WB, FCM, ELISA<br>Caspase 3 (WB 1:1000), Proteintech, Cat# 19677-1-AP; Species: Human, Mouse, Rat; Apply to IF, IHC, IP,WB, FCM, ELISA<br>Qki (WB 1:1000), CST, Cat# 86397; Species: Human, Mouse, Rat, Monkey; Apply to WB<br>NeuroD1 (WB 1:1000), Abcam, Cat# ab109224; Species: Mouse, Human; Apply to ICC, WB<br>cleaved-Caspase 3 (WB 1:1000), CST, Cat# 9664; Species: Human, Mouse, Rat, Monkey; Apply to IF, IP, IHC,WB, FCM<br>FUS (WB 1:1000; IF 1:100), CST, Cat# 67840; Species: Human, Mouse, Rat; Apply to IF, IP, WB<br>Nkx6.1 (WB 1:1000), CST, Cat# 54551; Species: Human, Mouse, Rat; Apply to IF, IP, IHC, WB, FCM<br>MafA (WB 1:1000), CST, Cat# 79737; Species: Human, Mouse; Apply to IF, IP, CHIP, WB<br>Pdx1 (WB 1:1000), CST, Cat# 5679; Species: Human, Mouse, Rat; Apply to IF, IP, WB<br>Scotin (WB 1:1000; IF 1:100), Sigma; Cat# HPA042295; Species: Human; Apply to IHC<br>Insulin (Rabbit, IF 1:200), CST, Cat# 3014; Species: Human, Mouse, Rat; Apply to IF, IHC, FCM<br>Insulin (Mouse, IF 1:200), CST, Cat# 8138; Species: Human, Mouse; Apply to IF, IP, WB

Eukaryotic cell lines

Policy information about cell lines and Sex and Gender in Research

Cell line source(s)	MIN6 cell was donated by Defu Zeng, Professor, from Departments of Diabetes Immunology and Hematopoietic Cell Transplantation Irell & Manella Graduate School of Biological Sciences of City of Hope, which original commercial source are obtained form ATCC (BCRJ).
Authentication	Human primary islets from Tianjin First Central Hospital.<br>Primary islet cells were isolated from mouse pancreas by collagenase digestion followed by Histopaque density gradient and manually picked up under microscope. Primary islet cells were stained dithizone to authenticate.<br>The mice pancreatic !-cell line MIN6 was donated by Defu Zeng professor, MIN6 cells were authenticated by qRT-PCR,WB, IF and ELISA assays. qRT-PCR and IF were used to test insulin gene expression, and ELISA assays was used to quantify insulin secretion.
Mycoplasma contamination	Cell lines were not tested for mycoplasma contaminatin.
Commonly misidentified lines<br>(See ICLAC register)	MIN6 cells, the mice pancreatic !-cell line, can synthesis insulin and secrete insulin. Thus, we choose MIN6 cells to mimic the primary islet cells. In this study, no commonly misidentified cell lines are used.

Animals and other research organisms

Policy information about studies involving animals; ARRIVE guidelines recommended for reporting animal research, and Sex and Gender in Research

Laboratory animals	All mice were housed 3–5 animals per cage, maintaining on a 12-h light and dark cycle with free access to water, at room temperature (25 ℃) and in the controlled humidity (60 ± 10 %).<br>C57BL/6J male mice, Lep ob/ob and Lepr db/db male mice were used in the study.<br>C57BL/6J mice were fed High Fat Diet (HFD) (D12494, 60% energy from fat) according to the criteria defined by Peyot ML, weighted between 45 and 50 g. The control groups were fed with normal diet (D12450J, 10% energy from fat), weighted between 23 and 25 g.<br>In this study, 8 weeks C57BL/6J male mice are injected adenovirus-circGlis3 (oe-circGlis3 mice), lentivirus-miR-124-3p (oe-miR-124-3p mice), lentivirus-Fus (oe-Fus mice), lentivirus-Scotin (oe-Scotin mice), adenovirus-circGlis3 and lentivirus-miR-124-3p (oe-circGlis3+ oe-miR-124-3p mice) , adenovirus-circGlis3 and lentivirus-Scotin (oe-circGlis3 + oe-Scotin mice), adenovirus-circGlis3 and lentivirus-Fus (oe-circGlis3 + oe-Fus mice). each group contain 12 male C57BL/6J mice.<br>Lepr db/db male mice in this study are injected adenovirus-circGlis3 (oe-circGlis3 mice) and adenovirus-control (oe-vector mice) at the age of 5 weeks. Each group contain 6 male C57BL/6J mice.
Wild animals	No wild animals were used in the study.
Reporting on sex	Only male mice were used in the study. Since female mice must be tested across the estrous cycle and are more variable than males, male mice were used in this proof-of-concept study.
Field-collected samples	No field-collected samples were used in the study.
Ethics oversight	All care and handling of animals were carried out according to the international laws and policies (EEC Council Directive 86/609, 1987) and approved by the animal ethics committee of China Pharmaceutical University (Nanjing, China) Care of animals was within institutional animal-care committee guidelines.

Note that full information on the approval of the study protocol must also be provided in the manuscript.

Flow Cytometry

Plots

Confirm that:

- [x] The axis labels state the marker and fluorochrome used (e.g. CD4-FITC).
- [x] The axis scales are clearly visible. Include numbers along axes only for bottom left plot of group (a 'group' is an analysis of identical markers).
- [x] All plots are contour plots with outliers or pseudocolor plots.
- [x] A numerical value for number of cells or percentage (with statistics) is provided.

Methodology

Sample preparation	For flow cytometry analysis of cell apoptosis, MIN6 cells (1×106 cells/well) were harvested and treated without EDTA, according to the instructions of Annexin V-FITC Apoptosis detection kit (Vazyme, Nanjing, china). After the double staining with FITC-Annexin V and Propidium iodide (PI), the cells were analyzed with a flow cytometry (FACScan®; BD Biosciences) equipped with FlowJo v10 software (BD Biosciences).<br>For flow cytometry Sorting of β-Cell and α-Cell, The islets were isolated and digested into single cells with 0.125% trypsin solution (without EDTA). The single cells were fixed in fixative solution (flow cytometry, Shanghai Huzheng) and permeabilized with permeabilization wash buffer (Yeasen). After preincubation with the blocking solution (20% mouse serum) at 4°C overnight, mouse anti-insulin (1: 100) and rabbit anti-glucagon (1:100) were both added to the cells and incubated at 37°C for 1 h. Goat anti-mouse Alexa Fluor 488 (1: 500) and donkey anti-rabbit Alexa Fluor 647 (1: 500) were both added to the cells. The cells were sorted by using BD FACSAria II SORP, and gating was performed using BD FACSDiva™ software (Becton, Dickinson Biosciences). Cell sorting was performed with a 100 μm nozzle size and sorted directly into 5 mL tubes containing 3 mL of PBS to minimize cellular stress. Cells (10,000~100,000) of each population of interest were sorted at a speed of 1500 cells/s.
Instrument	State Key Laboratory of Natural Medicines, Jiangsu Key Laboratory of Druggability of Biopharmaceuticals, School of life Science and Technology, China Pharmaceutical University.
Software	FlowJo 10 and BD Accuri C6 software.<br>BD FACSDiva software
Cell population abundance	For flow cytometry analysis of cell apoptosis, 100000 cells per well or group were digested and performed for the experiments. After digest, staining, washing , and cell flitration, about 50000 cells left at each sample.<br>For flow cytometry Sorting of β-Cell and α-Cell, 100000 cells per well or group were digested and performed for the experiments. After digest, staining, washing , cell flitration, and cell sorting, about 50000 cells left at each sample. The surviving cells were corrected into clean tubes, resuspended in 1 mL of TRIzol and prepared for RNA extraction.

Gating strategy

For flow cytometry analysis of cell apoptosis, the preliminary pi/FITC gates of the sorting cell population. Apoptosis cells were both positive in PI and FITC.
For flow cytometry Sorting of β-Cell and α-Cell, we stained cells with mouse anti-Insulin (IF 1:100), rabbit anti-glucagon (IF 1:100), Goat anti-mouse IgG H&L (Alexa Fluor® 488) (IF 1:500) and donkey anti-rabbit Alexa Fluor 647 (1: 500). The gating strategy was that chosen the obvious population.

☒ Tick this box to confirm that a figure exemplifying the gating strategy is provided in the Supplementary Information.
